# Rice Husk‐Derived Activated Carbons for Adsorption of Phenolic Compounds in Water

**DOI:** 10.1002/gch2.201800043

**Published:** 2018-10-25

**Authors:** Yafei Shen

**Affiliations:** ^1^ School of Environmental Science and Engineering Nanjing University of Information Science and Technology (NUIST) Nanjing 210044 P. R. China

**Keywords:** activated biocarbons, KOH activation, phenol adsorption, pyrolysis

## Abstract

Activated carbons are synthesized from rice husk by one‐ and two‐step pyrolysis. In general, two‐step pyrolysis produces a higher yield of activated carbons. The yield of activated carbon decreases with the increase of mass ratio of KOH and biomass, which has a significant impact on the development of surface area and porosity. The maximum *S*_BET_ (2138 m^2^ g^−1^) is achieved with micro‐ and mesoporous structures, which is favored for the adsorption process. The activated carbons can efficiently remove phenol from water by a few minutes. In particular, the maximum adsorption capacity (201 mg g^−1^) is achieved due to the excellent surface textural properties. The Langmuir model can better define the adsorption isotherm. The high correlation coefficient value (*R* ^2^ = 0.9991) indicates a monolayer adsorption behavior. The adsorption process can be well‐fitted by the pseudo‐second‐order model. Herein, the phenol molecules pass into the internal surface via liquid‐film‐controlled diffusion, so the behavior of phenol adsorption onto activated carbons is mainly controlled via chemisorption. In addition, the functional groups on the outer surfaces of activated carbons can attract the phenol molecules onto their internal surface via the “π–π dispersion interaction” and “donor–acceptor effect.”

## 1 Introduction

Rice husk (RH) as an abundant biowaste with low combustion value and damage to the environment can bring the issue of its sustainable utilization.^1^ However, RH can be converted to biofuels (e.g., syngas) through the pyrolysis at high temperatures.^2^, ^3^, ^4^, ^5^ RH is mainly composed of lignin (20–30%), holo‐cellulose (55–65%), SiO_2_ (15–20%), and extracts (2–5%), which can be regarded as a natural organic–inorganic composite.^6^, ^7^ Therefore, carbonization or activation of the lignin‐rich biomass (e.g., RH) in an inert atmosphere can yield highly porous carbons with large specific surface areas.^8^, ^9^ The conventional activation includes physical activation,^10^, ^11^ chemical activation,^12^ and integrated process.^13^ Additionally, the self‐activation using the gases emitted from biomass pyrolysis not only saves the cost of activating agents, but also decreases the environmental impact.^14^ In the physical activation, biochar is activated at a very high temperature (>900 °C) in the presence of steam or carbon dioxide. The chemical activation involves the impregnation of activating agents, such as KOH, K_2_CO_3_, ZnCl_2_, and H_3_PO_4_ with the biochar followed by activation in an inert atmosphere.^13^, ^15^ The latter is more efficient due to its lower consumption of energy and time. In particular, the chemical activation with KOH has been extensively developed for production of activated biocarbons.^16^, ^17^, ^18^

The KOH activation is a compelling method for creating highly microporous structure and functional groups on the char surface due to the intercalation of K between the lattices, joint oxidation of carbon and activation of carbon with in situ formed CO_2_.^19^, ^20^, ^21^ The KOH activation process generally undergoes two steps, including activation of biomass with KOH solution and subsequent carbonization at elevated temperature.^20^, ^21^ Herein, the activation process can be simplified by replacing the liquid KOH with the solid KOH. Up to date, the comparative study has been rarely reported on synthesis of activated biocarbons via the one‐ and two‐step pyrolysis process. The one‐step pyrolysis refers to copyrolysis of biomass with the activating agents (i.e., in situ activation), while the two‐step pyrolysis refers to activation of biochar with the activating agents (i.e., ex situ activation).

Phenolic compounds are the main components of tar.^22^, ^23^, ^24^ As a semivolatile organic compound (SVOC), it is difficult to degrade in nature. Significantly, human poisoning of phenol is mainly caused by contacting with respiratory tract and skin.^25^ The low concentration can induce protein denaturation, and the high concentration causes protein precipitation, thereby leading to a direct damage to cells and paralysis of central nervous system.^26^ Effective removal of phenol has been a significant issue in energy application and environmental protection. This work aims to comparatively study the synthesis of activated biocarbons from RH via one‐ and two‐step pyrolysis. And the physiochemical properties of activated carbons including textural properties, specific surface areas, and functional groups were characterized. Subsequently, the activated carbons were evaluated for adsorption of phenol.

## 2 Results and Discussion

### 2.1 Yield of Activated Biocarbons

**Figure 1:**
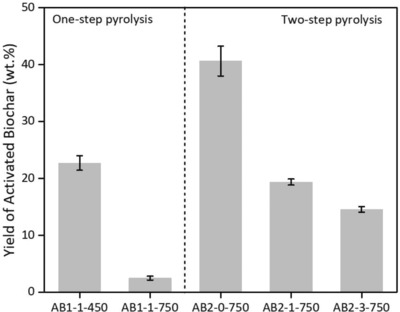
Yields of the activated biocarbons.

The yields of activated biocarbons are shown in **Figure** **1**. It can be seen that the yield of activated biocarbon decreased with the increase of the pyrolysis temperature. Two‐step pyrolysis can produce high yields of the activated biocarbons compared to one‐step pyrolysis. At 750 °C, the yield of AB2‐1‐750 (19.4%) is higher than that of AB1‐1‐750 (2.5%). Moreover, the yield of the activated biocarbon decreased with the increase of the mass ratio of KOH and RH. A highest yield (41%) of biochar can be achieved in AB2‐0‐750. It may be caused by the enhancement of KOH for carbon decomposition (Equations (1)–(4)).^27^ KOH and K_2_CO_3_ had significant effects on developing the surface area and porosity of carbon at different activation temperatures^27^, ^28^

(1) $$6\mathrm{KOH}+C\to 2K+2K_{2}{\mathrm{CO}}_{3}+3H_{2}$$

(2) $$K_{2}{\mathrm{CO}}_{3}+2C\to 2K+3\mathrm{CO}$$

(3) $$K_{2}{\mathrm{CO}}_{3}\to K_{2}O+{\mathrm{CO}}_{2}$$

(4) $$K_{2}O+C\to 2K+\mathrm{CO}$$

### 2.2 Characterization of Activated Biocarbons

#### 2.2.1 N_2_‐Sorption Isotherm

**Figure 2:**
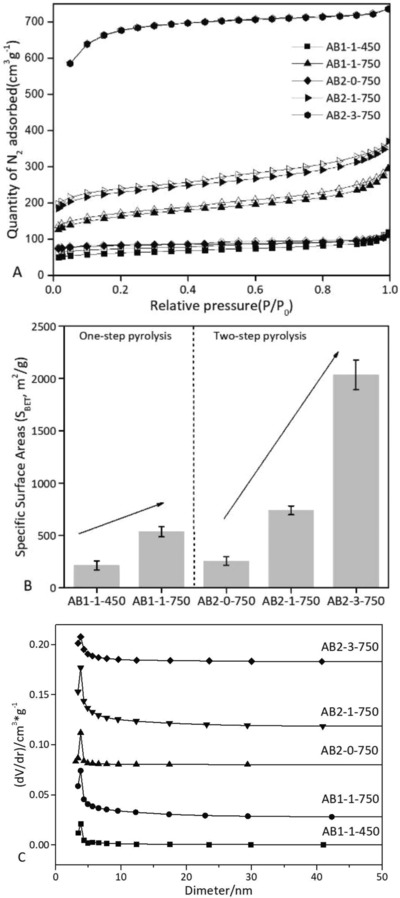
A) N_2_ adsorption–desorption isotherm, B) specific surface area (*S*_BET_), and C) pore size distribution of the activated biocarbons.

The process of adsorption is studied via graphs known as adsorption isotherm. It is the graph between the amounts of adsorbate (*x*) adsorbed on the surface of adsorbent (*m*) and pressure (*P*) at a constant temperature. The adsorption isotherms include Freundlich, Langmuir, and Brunauer–Emmett–Teller (BET) theory. The N_2_‐sorption isotherm is extensively applied for characterization of surface areas and pore structures.^29^ As shown in **Figure** **2**A, AB2‐3‐750 showed the type II adsorption isotherm. Under the lower relative pressure (*P*/*P*_0_), the adsorption capacity increased rapidly, indicating that the mesoporous carbon possessed many internal microporous structures. The adsorption capacity can increase with the increase of the relative pressure, but the hysteresis loop was not observed remarkably. Additionally, AB1‐1‐450 and AB2‐0‐750 were the type II adsorption isotherms. It indicated the samples presented many mesopores. However, AB1‐1‐750 and AB2‐1‐750 were type IV without the remarkable hysteresis loop. Accordingly, the calculated specific surface areas (*S*_BET_) are given in Figure 2B. It could be found that AB2‐3‐750 had the highest specific surface area (*S*_BET_ = 2138 m^2^ g^−1^). In general, the specific surface area increased with the increase of pyrolysis temperature and KOH amount. Furthermore, the pore size distribution was obtained (Figure 2C). The pores in activated biocarbons were the microporous and mesoporous structures (mostly <10 nm). These results indicated that the activated biocarbons were favorable for the enhancement of adsorption capacity by providing sufficient adsorption sites.

#### 2.2.2 Scanning Electron Microscope (SEM) Analysis

**Figure 3:**
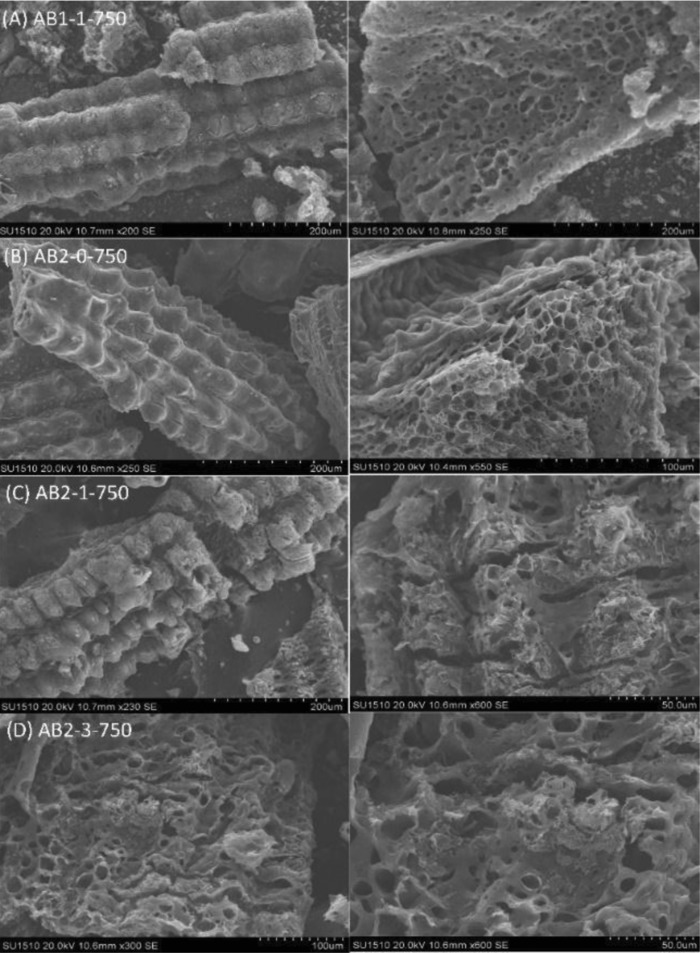
A–D) SEM images of AB1‐1‐750, AB2‐0‐750, AB2‐1‐750, and AB2‐3‐750.

The microstructures of activated biocarbons were characterized by SEM analysis. As shown in **Figure** **3**A, the outer epidermis of RH char derived from one‐step pyrolysis was covered with a uniform corrugated structure like the original RH. It was so‐called the “ascular bundle structure” with nanosilica on the surface, which acted as a natural template for the synthesis of porous carbons.^6^ With the increase of the pyrolysis temperature and time, the ridgy structure became more obvious (Figure 3B). Without the KOH activation, AB2‐0‐750 also showed abundant porous structures on the biochar surface (Figure 3B), most likely caused by the devolatilization of RH. However, the specific surface area of AB2‐0‐750 was still low without the KOH activation to form the new pores.

Indeed, the biochar can be further decomposed using the KOH activation acutely via two‐step pyrolysis (Figure 3C). It was seen that many new macropores were formed on the outer surface of biocarbons (Figure 3C,D). Consequently, these macropores contributed to the formation of new meso‐ and micropores in the internal surface. Hence, the interconnected open meso‐ and microporous structures can promote the mass transfer of phenol, resulting in the improvement of adsorption capacity.

#### 2.2.3 IR Analysis

**Figure 4:**
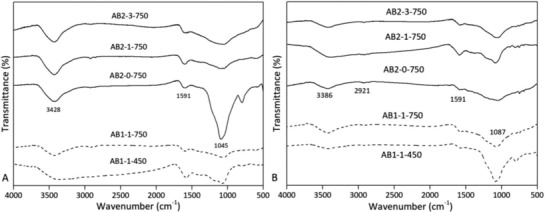
A,B) IR spectrum of the activated biocarbons before and after use.

Besides the porosity properties, the configuration of functional groups on the surface is considered another important factor that determines the adsorption behavior of the porous carbon adsorbents. The IR spectrum of activated biocarbons in **Figure** **4**A showed the stretching vibrations of O—H and N—H (3428 cm^−1^), C—H (2921 cm^−1^), C=O and C=N (1591 cm^−1^), and C—O (1045 cm^−1^). In particular, the out‐of‐plane vibration of —COOH (carboxylic acid) occurred at 799 cm^−1^. It may be attributed to the formation of carboxylic acids during the RH pyrolysis. Meanwhile, the O—H, C=O, and C—O groups are probably contributed from the etching effect of KOH on porous carbon networks during the activation process.^29^ After the adsorption process, the surface functional groups had no obvious changes (Figure 4B). Small peaks at 670 cm^−1^ occurred, indicating the bending vibration of C—H bond. In addition, the O—H bond at 3386 cm^−1^ appeared a weak vibration. These results suggested that the adsorbed phenol can influence the functional groups on the surface.

### 2.3 Adsorption Capacity

**Figure 5:**
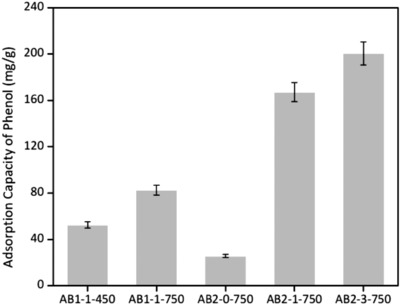
Adsorption capacity of phenol with activated biocarbons.

To evaluate the performance of activated biocarbons on the adsorption of phenol, the pH value (7), initial phenol concentration (0.5 g L^−1^, 50 mL) and contact time (4 h) were confirmed. In general, the adsorption capacity was associated with *S*_BET_. As shown in Figure 8, the maximum adsorption capacity (201 mg g^−1^) was achieved with AB2‐3‐750. Moreover, AB2‐1‐750 with relatively low *S*_BET_ had a higher adsorption capacity of 167 mg g^−1^ (**Figure** **5**). It was attributed to microporous structures to host the adsorbed phenol molecules. The existence of surface carbonyl and pyrrolic‐N groups may attract the phenol molecules onto the internal surfaces of biocarbons via the “π–π dispersion interaction” and “donor–acceptor effect.”^30^

### 2.4 Adsorption Kinetics

**Figure 6:**
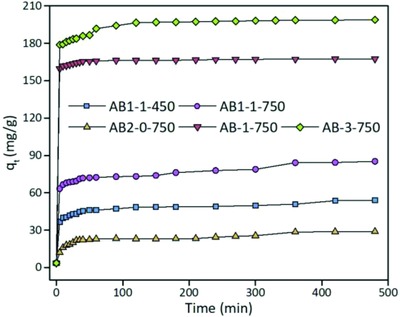
Effect of contact time on the adsorption capacity.

The adsorption equilibrium was also investigated for phenol removal by the activated biocarbons. As shown in **Figure** **6**, the activated biocarbons from RH can efficiently remove phenol from wastewater. The adsorption processes were completed through several minutes. Among them, AB2‐1‐750 and AB2‐3‐750 exhibited the highest adsorption rates and capacities for phenol removal. The adsorption capacity reached about 160 and 179 mg g^−1^, respectively, only by 5 min. It was contributed to their excellent surface porosity properties along with higher specific surface areas. AB1‐1‐450 and AB2‐0‐750 had a similar *S*_BET_, but the adsorption capacity of the former was much higher than that of the latter (nearly two times). Also, AB1‐1‐450 exhibited more functional groups by pyrolysis at a lower temperature. It indicated that the functional groups such as —OH and —NH can enhance the adsorption process on the surface of biocarbon.

**Figure 7:**
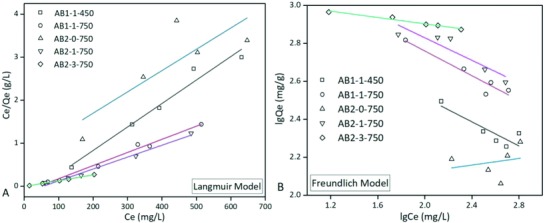
A,B) Adsorption isotherm linear fitting by Langmuir and Freundlich models.

The Langmuir and Freundlich isotherm models have been widely applied to describe the adsorption behavior of phenol on activated carbons.^32^ The linear fitting results of adsorption isotherms are depicted in **Figure** **7** and the parameters are given in Table S1 (Supporting Information). Herein, the Langmuir model (Figure 7B) can well describe the adsorption isotherms. Particularly, AB2‐3‐750 with relatively high correlation coefficient value (*R* ^2^ = 0.9991) indicated a monolayer adsorption behavior.^29^ Both the increase of pyrolysis temperature and KOH activation enhanced the surface heterogeneity of biochars.^31^

**Figure 8:**
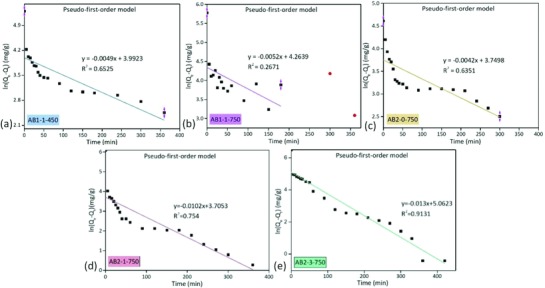
Adsorption kinetics fitted by the pseudo‐first‐order model.

**Figure 9:**
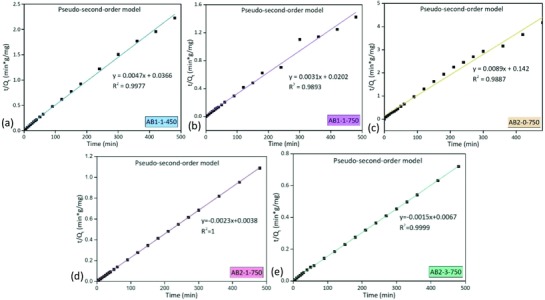
Adsorption kinetics fitted by the pseudo‐second‐order model.

Currently, the adsorption kinetics were investigated by the pseudo‐first‐order (8) and pseudo‐second‐order (9) models. As shown in **Figures** **8** and **9**, *R* ^2^ indicated that the adsorption process of phenol onto the activated biocarbons could be well described by the pseudo‐second‐order model with a much larger *R* ^2^ than those of the pseudo‐first‐order model. The phenol molecules passed into the internal char surface through the liquid‐film‐controlled diffusion, so the behavior of the phenol adsorption onto activated biocarbons was mainly controlled via the chemisorption.^32^ However, Zhang et al.^33^ studied the behavior of phenol adsorption on the activated carbons. Thermal modification for activated carbons at 900 °C can result in the decrease of pore morphology and oxygen‐containing functional groups (e.g., phenolic hydroxyl, lactone base, and carboxyl). The maximum adsorption capacity reached 144.93 mg g^−1^. Since the pseudo‐second‐order kinetic and Langmuir model can fit the data well, the phenol adsorption on the modified activated carbons was an exothermic process and mainly via physisorption.^33^ Since the activated biocarbons possessed a variety of functional groups on the inner and outer surfaces, the interaction of hydrogen bonds between phenol and functional groups can explain the adsorption process as well.

## 3 Conclusions

The activated biocarbons from RH were synthesized by one‐ and two‐step pyrolysis. Two‐step pyrolysis could produce a higher yield of activated biocarbon. At 750 °C, the yield of AB2‐1‐750 (19.4%) was much higher than that of AB1‐1‐750 (2.5%). The yield of activated biocarbon decreased with the increase of the mass ratio of KOH and biomass, which had a significant impact on developing surface area and porosity of carbon at different activation temperatures. Additionally, the activated biocarbon (e.g., AB2‐3‐750) had the highest specific surface area (*S*_BET_ = 2138 m^2^ g^−1^) along with the micro‐/mesoporous structures. The activated biocarbons can efficiently remove phenol from water. The adsorption process was completed by only a few minutes. In particular, the maximum adsorption capacity of 201 mg g^−1^ was achieved using AB2‐3‐750. It was attributed to the excellent surface porosity properties with higher surface areas. With regards to the phenol adsorption onto the activated biocarbons, the Langmuir models can well define the adsorption isotherms. AB2‐3‐750 with a higher correlation coefficient value (*R* ^2^ = 0.9991) indicated a monolayer adsorption behavior. Also, the adsorption process can be well fitted by the pseudo‐second‐order model (*R* ^2^ = 1). The phenol molecules passed into the internal surface through the liquid‐film‐controlled diffusion. Therefore, the behavior of phenol adsorption onto activated carbon was mainly controlled via chemisorption. In addition, the functional groups on the outer surfaces of activated carbons can attract the phenol molecules onto their internal surfaces via the “π–π dispersion interaction” and “donor–acceptor effect.”

## 4 Experimental Section

*Feedstocks and Chemicals*: The feedstock of RH was collected from a rice‐milling factory in Jiangsu (China). The original RH was washed out by the distilled water to remove the impurities. Then, it was dried in an oven at 105 °C overnight. The clean RH was dry stored for further use. The properties of RH mainly including proximate analysis and ultimate analysis are shown in **Table** **1**. The chemicals including KOH (AR, >99.5%) and phenol (AR, >99%) were purchased from Sinophram (China).

**Table 1:** Properties of the RH feedstock

Ultimate analysis [wt%, dry and ash free]				Proximate analysis [wt%, dry]			HHVa) [MJ kg^−1^]
C	H	Ob)	N	VMc)	FCd)	Ash	
44.00	5.20	50.38	0.42	54.38	22.58	23.04	16.05
Chemical composition [wt%]							
SiO_2_	Al_2_O_3_	Fe_2_O_3_	CaO	MgO	Na_2_O	K_2_O	ZnO
94.64	0.06	0.23	1.38	0.96	0.39	0.58	0.01

^a)^ HHV (higher heating value) = 0.1905[VM] + 0.2521[FC]

^b)^ Calculated by mass difference

^c)^ VM: volatile matters

^d)^ FC: fixed carbon.

*Synthesis of Activated Biocarbons*: The pyrolysis experiments were conducted in a fixed‐bed reactor mainly including a furnace coupled with an electric controller, a cooling system, a liquid collector, and a gas collection system. For the synthesis of activated biocarbon by one‐step pyrolysis, RH blended with the solid KOH (10 g, mass ratio: 1) without milling was initially fed into the furnace. Before heating, N_2_ was continuously introduced to the reactor with a flow rate of 1.0 L min^−1^ for 30 min to remove the air. The final temperature was controlled at 450 or 750 °C (heating rate: 20 °C min^−1^) with a retention time of 3 h to ensure the complete pyrolysis. As the entire process was finished, the products of char, oil, and gas were collected. The char was washed out by the distilled water till the pH value of the filtrate was close to 7. After that, the activated biocarbons with neutral properties were dried in an oven at 120 °C for 2 h. Finally, the dry activated biocarbons defined as AB1‐1‐450 and AB1‐1‐750 were measured by weighing and stored for further use.

For the two‐step pyrolysis, RH (5 g) was pyrolyzed at 420 °C under the N_2_ (heating rate: 20 °C min^−1^; retention time: 3 h) to produce the biochar. After that, the biochar mixed with the solid KOH (mass ratio: 0, 1, and 3) without milling were activated in the furnace at 750 °C (heating rate: 20 °C min^−1^; retention time: 1 h). The activated biocarbons were washed out by the distilled water for several times till the pH value of the filtrate was 7. The activated biocarbons with neutral properties were dried at 120 °C for 2 h. Finally, the dry activated biocarbons defined as AB2‐0‐750, AB2‐1‐750, and AB2‐2‐750 were measured by weighing and stored for use.

*Adsorption Tests and Kinetic Studies*: The plenty of phenol solution (0.5 g L^−1^) was initially prepared for the adsorption experiments. For each test, the activated biocarbon (0.1 g) was used for adsorption of phenol (0.5 g L^−1^, 50 mL) in the beaker (100 mL) at room temperature (≈20 °C). By magnetically stirring (1000 rpm) of the beaker for a designed time (5–480 min), the liquid and solid phases were separated by the vacuum filtration. And the supernatant liquid was sampled to determine its absorbance using the UV–visible spectroscopy. All measurements were made at a specific wavelength (269.5 nm) of phenol at 25 °C. Thus, the correlation between absorbance and the concentration of phenol was determined using the Lambert–Beer's law. Each test was conducted in triplicates and the average result was used.

The adsorption capacity (*q*_e_) at equilibrium was determined using Equation (5)

(5) $$q_{e}=\frac{\left(C_{0}-C_{t}\right)V}{m}$$

The Langmuir (6) and Freundlich (7) model equations were used to fit the data to describe the adsorption equilibrium of activated biocarbons^34^

(6) $$q_{e}=q_{m}\;\times b\times C_{e}/\left(1+b+C_{e}\right)$$

(7) $$q_{e}=K_{F}\;\times C_{e}^{1/n}$$

where *C*_e_ (mg L^−1^) is the equilibrium concentration of phenol, *q*_e_ (mg g^−1^) is the amount of phenol adsorbed, *q*_m_ (mg g^−1^) is the saturate adsorption capacity of phenol, *b* (L mg^−1^) is the Langmuir isotherm coefficient, *K*_F_ (mg g^−1^) and n are the Freundlich constants.

*Adsorption Kinetics*: The adsorption kinetics of phenol with the activated biocarbons were calculated by the pseudo‐first‐order and pseudo‐second‐order kinetic Equations (8) and (9), respectively^35^

(8) $$\ln\left(q_{e}-q_{t}\right)=\;\ln Q_{e}-tK_{1}$$

(9) $$t/q_{t}=1/\left(K_{2}q_{e}^{2}\right)+t/q_{e}$$

where *K*_1_ (min^−1^) is the rate constant of the pseudo‐first‐order, *K*_2_ (min^−1^) is the rate constant of the pseudo‐second‐order, and *t* (min) is the adsorption time.

*Analytical Methods*: The proximate analysis was performed by the thermogravimetric analysis (TGA‐4000, PerkinElmer, USA). The ultimate analysis was performed by the element analyzer (EA, VARIO EL III, Element, Germany). The chemical constitutes of ash were determined by the X‐ray fluorescence (XRF, Olympus Innovex X‐5000, Canada). The specific surface area was determined using the BET method according to the N_2_‐sorption isotherm. The surface functional groups were characterized by the infrared spectrometer (IR, Avatar360, Nicolet, USA). The surface microstructures were analyzed by SEM (SU1510, Hitachi, Japan). The crystal structure was analyzed by X‐ray diffraction (XRD‐6100, Shimadzu, Japan). In addition, the phenol concentration in the liquid was determined using the UV–visible spectroscopy (UV2401, Shimadzu, Japan).

## Conflict of Interest

The authors declare no conflict of interest.

## Supporting information

Supplementary
