# Supplementary material for: Rice Husk‐Derived Activated Carbons for Adsorption of Phenolic Compounds in Water
Source: Glob Chall. 2018 Oct 25;2(12):1800043. doi: 10.1002/gch2.201800043 (PMC6607306; doi:10.1002/gch2.201800043)
Supplement: Supplementary file 1 — Supplementary [file GCH2-2-1800043-s001.pdf]

Copyright WILEY-VCH Verlag GmbH & Co. KGaA, 69469 Weinheim, Germany, 2018.

Global Challenges
Open Access

Supporting Information

for *Global Challenges,* DOI: 10.1002/gch2.201800043

Rice Husk-Derived Activated Carbons for Adsorption of Phenolic Compounds in Water

Yafei Shen

Table S1 Parameters of adsorption isotherms

Activated Bio-carbons		200	400	500	600	800
AB1-1-450	C_e	137.44	313.15	403.21	491.80	630.45
	C_e/Q_e	0.44	1.44	1.82	2.73	3
	Langmuir	R^2=0.9688				
AB1-1-750	C_e	68.48	214.74	329.59	364.37	514.13
	C_e/Q_e	0.1	0.46	0.97	0.93	1.44
	Langmuir	R^2=0.9846				
AB2-0-750	C_e	168.98	345.6	442.47	503.09	647.13
	C_e/Q_e	1.09	2.54	3.85	3.11	3.39
	Langmuir	R^2=0.6901				
AB2-1-750	C_e	59.34	128.36	165.17	323.94	484.44
	C_e/Q_e	0.08	0.19	0.25	0.7	1.23
	Langmuir	R^2=0.9873				
AB2-3-750	C_e	15.23	52.96	102.35	129.1	203.09
	C_e/Q_e	0.016	0.061	0.13	0.1644	0.27
	Langmuir	R^2=0.9991				

Activated Bio-carbons		200	400	500	600	800
AB1-1-450	lgC_e	2.14	2.5	2.6	2.69	2.8
	lgQ_e	2.5	2.34	2.29	2.26	2.33
	Freundlich	R^2=0.7637				
AB1-1-750	lgC_e	1.84	2.33	2.52	2.56	2.71
	lgQ_e	2.82	2.67	2.53	2.59	2.55
	Freundlich	R^2=0.9144				
AB2-0-750	lgC_e	2.23	2.54	2.65	2.9	2.81
	lgQ_e	2.19	2.13	2.06	2.21	2.28
	Freundlich	R^2=0.0549				
AB2-1-750	lgC_e	1.77	2.11	2.22	2.51	2.69
	lgQ_e	2.85	2.83	2.83	2.66	2.6
	Freundlich	R^2=0.8397				
AB2-3-750	lgC_e	1.18	1.72	2.01	2.11	2.31
	lgQ_e	2.97	2.94	2.9	2.89	2.87
	Freundlich	R^2=0.9626				
